# The Effect of Cholesterol Content on the Adjuvant Activity of Nucleic-Acid-Free Lipid Nanoparticles

**DOI:** 10.3390/pharmaceutics16020181

**Published:** 2024-01-26

**Authors:** Jessica Anindita, Hiroki Tanaka, Takuma Yamakawa, Yuka Sato, Chika Matsumoto, Kota Ishizaki, Taiji Oyama, Satoko Suzuki, Keisuke Ueda, Kenjirou Higashi, Kunikazu Moribe, Kasumi Sasaki, Yumika Ogura, Etsuo Yonemochi, Yu Sakurai, Hiroto Hatakeyama, Hidetaka Akita

**Affiliations:** 1 Laboratory of DDS Design and Drug Disposition, Graduate School of Pharmaceutical Sciences, Tohoku University, 6-3 Aoba Aramaki, Aoba-ku, Sendai City 980-8578, Miyagi, Japan; 2 Laboratory of DDS Design and Drug Disposition, Graduate School of Pharmaceutical Sciences, Chiba University, 1-8-1 Inohana, Chuo-ku, Chiba City 260-0856, Chiba, Japan; 3 Center for Advanced Modalities and DDS, Osaka University, Suita 565-0871, Osaka, Japan; 4 Sales Division, JASCO Corporation, 2967-5 Ishikawa, Hachioji City 192-8537, Tokyo, Japan; taiji.oyama@jasco.co.jp; 5 Applicative Solution Lab Division, JASCO Corporation, 2967-5 Ishikawa, Hachioji City 192-8537, Tokyo, Japan; 6 Laboratory of Pharmaceutical Technology, Graduate School of Pharmaceutical Sciences, Chiba University, 1-8-1 Inohana, Chuo-ku, Chiba City 260-0856, Chiba, Japan; keisuke@chiba-u.jp (K.U.);; 7 Department of Physical Chemistry, School of Pharmacy and Pharmaceutical Sciences, Hoshi University, 2-4-41 Ebara, Shinagawa-ku, Shinagawa City 142-8501, Tokyo, Japan

**Keywords:** lipid nanoparticle, cholesterol, particle morphology, adjuvant activity

## Abstract

RNA vaccines are applicable to the treatment of various infectious diseases via the inducement of robust immune responses against target antigens by expressing antigen proteins in the human body. The delivery of messenger RNA by lipid nanoparticles (LNPs) has become a versatile drug delivery system used in the administration of RNA vaccines. LNPs are widely considered to possess adjuvant activity that induces a strong immune response. However, the properties of LNPs that contribute to their adjuvant activity continue to require clarification. To characterize the relationships between the lipid composition, particle morphology, and adjuvant activity of LNPs, the nanostructures of LNPs and their antibody production were evaluated. To simply compare the adjuvant activity of LNPs, empty LNPs were subcutaneously injected with recombinant proteins. Consistent with previous research, the presence of ionizable lipids was one of the determinant factors. Adjuvant activity was induced when a tiny cholesterol assembly (cholesterol-induced phase, ChiP) was formed according to the amount of cholesterol present. Moreover, adjuvant activity was diminished when the content of cholesterol was excessive. Thus, it is plausible that an intermediate structure of cholesterol (not in a crystalline-like state) in an intra-particle space could be closely related to the immunogenicity of LNPs.

## 1. Introduction

RNA vaccines that are based on in vitro-transcribed messenger RNA (IVT-mRNA) induce robust immune responses against specific antigens by expressing the antigen proteins in our bodies [1,2,3,4,5]. Since the targets of RNA vaccines can be readily modified by designing the sequence of the IVT-mRNA, RNA vaccines against various infectious diseases can be quickly developed based on genetic information. The intrinsic inflammatogenicity of IVT-mRNA is ameliorated by improvements in the quality of the IVT-mRNA (i.e., the replacement of Uridine nucleotides with chemically modified ones [6,7], the addition of a Cap1 structure to the 5′ end [8,9], and the removal of dsRNA contaminants) [10,11]. On the other hand, since the purpose of improving the IVT-mRNA was not to increase the chemical/biological stability of the IVT-mRNA, the IVT-mRNA remains vulnerable to degradation in the human body. Therefore, the therapeutic use of IVT-mRNA requires an efficient drug delivery system (DDS).

Currently, there is a series of DDS platforms such as polymer-based vectors [12,13] and lipid-based vectors [14,15]. Among these DDSs, lipid nanoparticles (LNPs) are common systems that have been approved for use in clinical situations. The main component of LNPs that is important for their function is ionizable lipids equipped with tertiary amine moieties. These LNPs are designed to have a neutral charge in a physiological environment in order to avoid an unspecific electrostatic interaction with biomaterials. In contrast, once they are taken up by cells via endocytosis, the amine moieties develop positive charges under an endosomal acidic environment (pH 5.5–6.5). The LNPs interact with the anionic endosomal membranes, which causes an endosomal degradation that promotes the endosomal escape of mRNA [16,17]. In addition to ionizable lipids, LNPs also contain phospholipids, sterols, and polyethylene glycol-conjugated lipids (PEG lipids) for stabilization [18,19].

Meanwhile, the COVID-19 pandemic caused by the SARS-CoV-2 virus has resulted in millions of hospitalizations and deaths worldwide. The outbreak was controlled by the development of LNP-based RNA vaccines by Moderna (Spikevax^®^) [20,21] and Pfizer/BioNTech (Comirnaty^®^) [22,23]. Given the significant vaccine efficacy of the mRNA-LNPs, it is plausible that mRNA-LNPs intrinsically possess “adjuvant activity”. However, the mechanism of the immune stimulation of mRNA-LNPs has not been clarified. IVT-mRNA loaded in mRNA-LNPs is a possible candidate for an activator of immune systems. Extracellular RNA molecules are generally recognized as signals of invasion by a pathogenic microorganism. RNAs, specifically unmodified RNAs, can be recognized by pattern recognition receptors (PRRs). PRRs, particularly Toll-like receptors (TLRs) such as TLR-3, TLR-7, and TLR-8, are capable of recognizing non-self RNAs in endosomes. Other PRR families, including retinoic acid-inducible gene-I (RIG-I) receptors and melanoma differentiation-associated gene 5 (MDA-5), are known to be capable of recognizing non-self RNAs in cytosol [24,25,26]. The recognition of RNAs by PRRs triggers the production of type-I interferons (IFN-1) such as IFNα [24,25,26,27]. MDA-5 receptors can be activated by modified RNAs and are known to be important for both IFN-I responses and for the activation of antigen-specific CD8+T cell responses [27,28]. Consistent with this notion, the IVT-mRNA encapsulated in mRNA-LNPs actually contributes to the adjuvant activity of mRNA-LNPs—even when these are chemically modified nucleotides.

On the other hand, LNPs are known to possess adjuvant activity even in the case of empty LNPs without IVT-mRNA [29]. Empty LNPs promote antibody production against co-injected antigen proteins on a level that is comparable to Alum-based adjuvants [30]. The differentiation and proliferation of follicular helper T cells and germinal center B cells are reproduced by the injection of a mixture of empty LNPs and recombinant antigen proteins [31]. A topical injection of empty LNPs induces the secretion of various chemokines and the infiltration of neutrophils and monocytes at the injection site [32]. These observations clearly suggest that LNPs have the ability to stimulate the immune system. Among the contents of LNPs, ionizable lipids are considered a cause of this immune stimulation. As far as we could ascertain, however, there is little evidence that ionizable lipids themselves are recognized as non-self-molecules by PRRs. Considering that various LNPs composed of different ionizable lipids have shown similar vaccine activities, the reported ionizable lipids could not be expected to stimulate receptors specific for each of them. Rather, it would be reasonable to assume that a characteristic common to various LNPs is responsible for their adjuvant activity.

The adjuvant activity of empty LNPs almost completely disappears when ionizable lipids are removed from their lipid compositions [31]. This observation has been a major basis for the hypothesis that ionizable lipids determine the adjuvant activity of LNPs. However, when ionizable lipids are removed from LNPs, the morphology of the particles can be drastically changed. Since ionizable lipids do not have a cationic charge in a physiological environment, these molecules show hydrophobic oil-like properties [33]. The removal of ionizable lipids induces structural changes in these nanoparticles that differ from those of nanoemulsion-like structures with hydrophobic cores and vesicle structures with lipid bilayers. When analyzing the biological responses to nanoparticles, such morphological changes in particles are also important [34,35,36,37]. In the present study, the effects that the lipid composition exert on the structural properties of LNPs were analyzed along with their adjuvant activity. We focused on the cholesterol content of LNPs, since a large portion of LNPs contain cholesterol or sterol derivatives as the second most common ingredient.

## 2. Materials and Methods

### 2.1. Animals

C57BL/6J mice (female, 6 weeks) were purchased from Nippon SLC, Inc. (Shizuoka, Japan). Protocols for the animal experiments were reviewed and approved by the Chiba University Animal Care Committee and Tohoku University Animal Care Committee following the “Guide for Care and Use of Laboratory Animals” (No. 4-175 492 (Chiba University) and No. 2021-011-04 (Tohoku University)).

### 2.2. Materials

A detailed list of supplier information including item numbers of all reagents used in this study appears in the Supplementary Materials (Table S1). The ionizable lipid SM-102 was purchased from Cayman Chemical (Ann Arbor, MI, USA). The 1,2-Distearoyl-sn-glycero-3-phosphocholine (DSPC, Product# COATSOME^®^ MC-8080) and 1-(Monomethoxy polyethyleneglycol2000)2,3-dimyristoylglycerol (DMG-PEG_2000_, Product# SUNBRIGHT^®^ GM-020) were purchased from NOF CORPORATION (Tokyo, Japan). Cholesterol was purchased from Sigma-Aldrich (St. Louis, MO, USA). The ovalbumin (OVA) protein (Product# Albumin from chicken white egg) was purchased from Sigma-Aldrich (St. Louis, MO, USA). All other reagents and chemicals are commercially available and were used without further purification.

### 2.3. Methods

#### 2.3.1. Preparation of LNPs

Three groups of particles were prepared in this study: dispersed cholesterol particles, liposomes, and LNPs. The dispersed cholesterol particles were composed of cholesterol/DMG-PEG 2000 = 98.5/1.5 mol%. The liposome particles (Liposome_A to Liposome_D) were composed of DSPC, cholesterol, and DMG-PEG_2000_. The LNP particles (LNP_A to LNP_J) were composed of SM-102, DSPC, cholesterol, and DMG-PEG_2000_. The details of each composition are elaborated in Table 1. The LNP_C is the original lipid composition used in RNA vaccines, and it is composed of SM-102/DSPC/Cholesterol/DMG-PEG 2000 = 50/10/38.5/1.5 mol% of total lipid [38,39]. The lipid mixtures in 200 nmol concentrations were dissolved in ethanol and combined with a 6.25 mM sodium acetate buffer (pH 5.0) [40] by using a NanoAssemblr^®^ Ignite™ device (Precision Nanosystems, Vancouver, Canada) with a flow rate setting of 2.0 mL/min, a flow rate ratio (buffer/lipid) of 3:1, a total volume of 1.3 mL, a start waste of 0.25 mL, and an end waste of 0.05 mL. The formulations were recovered and diluted with 20 mM MES buffer (pH 5.5). The formulations were concentrated with D-PBS (−) by ultrafiltration using Amicon Ultra Centrifugal Units (#UFC810096, #UFC910096, Merck, Rahway, NJ, USA). The LNPs were recovered and diluted to an adequate volume with D-PBS (−).

#### 2.3.2. Characterization of the LNPs

The LNPs were diluted (1000×) with D-PBS (−) for particle size and polydispersity index (PdI) analysis. The LNPs were diluted (30×) with 10 mM HEPES buffer (pH 7.4) for zeta-potential evaluation. Particle size, PdI, and zeta-potential of LNPs were determined using an electrophoretic light-scattering spectrophotometer (Zetasizer Nano ZS, Malvern Panalytical, Malvern, UK).

#### 2.3.3. OVA Antigen-Specific Total IgG Quantification

BALB/c mice were subcutaneously administered (back of neck) empty LNPs mixed with 10 µg of OVA protein in 100 µL D-PBS (−) on days 0 and 14, respectively. Blood collection was carried out on days 14 (before the second administration) and 28. Blood serum was obtained by incubating the blood at room temperature (RT) for 2 h, following centrifugation (4 °C, 2000× *g*, 10 min). The OVA antigen-specific antibody was quantified using an enzyme-linked immunosorbent assay (ELISA). Plate wells (clear flat-bottomed immuno non-sterile 96-well plates, Thermo Scientific, Waltham, MA, USA) were coated with 100 µL of OVA protein (10 µg/mL in 50 mM NaHCO_3_ pH 9.6) for 16 h at 4 °C. The plate was washed with 0.1% (*w*/*v*) Tween 20 in D-PBS (−) and then blocked with 5% (*w*/*v*) Fetal Bovine Serum (FBS, #10270, Gibco, New York, NY, USA) following incubation (37 °C, 2 h). Samples of blood serum were diluted with 0.1% (*w*/*v*) Tween 20 in D-PBS (−) and added into the wells at concentrations of 1/100 following incubation (RT, 1 h). The plate was washed, and a 1/2000 dilution of Goat anti-Mouse IgG-Fc Fragment Antibody HRP Conjugated (A90-131P, Bethyl Laboratories, Montgomery, TX, USA) was added into the wells following incubation (RT, 1 h). The plate was once again washed, and 100 µL of TMB Solution (CL07-100ML, Merck Millipore, Burlington, MA, USA) was added into each well. The plate was incubated in a dark environment (RT, 30 min), and then 100 µL of 1 M H_2_SO_4_ (stop solution) was added. The absorbances were measured using a plate reader (Infinite M200 PRO, TECAN, Männedorf, Switzerland) set to λ = 450 nm. The OVA antigen-specific total IgG levels were determined directly from the absorbance values.

#### 2.3.4. SAXS

Small-angle X-ray scattering (SAXS) measurements of the suspension of LNPs were conducted using the BL-10C beamline of the Photon Factory at the High Energy Accelerator Research Organization (Tsukuba, Japan). For the SAXS measurements, the external solution of the LNPs was replaced with nuclease-free water. The final concentration of the LNPs was 20 mM of total lipids. The wavelength and camera length were 0.9 Å and 50 cm, respectively. Diffraction images were analyzed using SAngler software (ver. 2.1.64) [41].

#### 2.3.5. Cryo-TEM

A Cryo-TEM image was obtained according to a previous report [42] using a JEM-2100F field-emission TEM apparatus (JEOL Co., Ltd., Tokyo, Japan) with an accelerating voltage of 120 kV. For Cryo-TEM, 2 μL of the nanoparticle suspension (40 mM total lipids in Nuclease free water) was deposited onto a 200-mesh copper grid covered with carbon film (Nisshin EM Co., Ltd., Tokyo, Japan). After removing the excess liquid using filter paper, the sample was rapidly vitrified by immersion in liquid ethane using a Leica CPC cryo-preparation chamber (Leica Microsystems, Wetzlar, Germany). The grid with the vitrified thin film was placed on a sample holder that was maintained below −170 °C using liquid nitrogen. Images were recorded using a CCD camera.

## 3. Results

### 3.1. Adjuvant Activity of the LNPs Did Not Depend on the Amount of Ionizable Lipids

**Figure 1:**
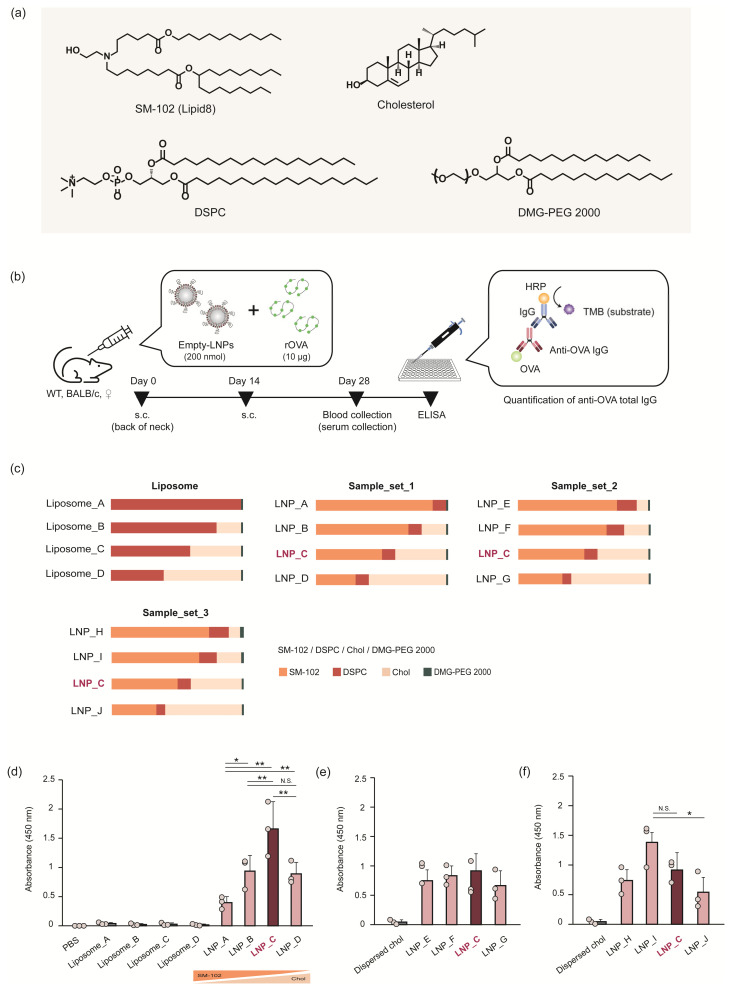
Schemes for the experiments and for antibody production. (**a**) Chemical structures of SM-102, DSPC, cholesterol, and DMG-PEG_2000_. (**b**) In the experimental design, BALB/c mice were injected subcutaneously (s.c.) with 200 nmol empty LNPs mixed with 10 µg recombinant OVA proteins. The administration was carried out twice with a 14-day interval. On day 28, the blood of each mouse was collected, and the serum was used for anti-OVA total IgG quantification by an ELISA. (**c**) Lipid compositions of particles used in this study are as follows: liposomes (Liposome_A–D), Sample_set_1 (LNP_A–D), Sample_set_2 (LNP_C, LNP_E–G), and Sample_set_3 (LNP_C, LNP_H–J). The results of antibody quantification are as follows: (**d**) liposomes, Sample_set_1, (**e**) Sample_set_2, and (**f**) Sample_set_3. Each LNP was independently injected into three mice (*n* = 3). The scatter plots represent the individual values; the bar graph represents the mean with SD (*n* = 3). N.S.: not significant; * *p* < 0.05, ** *p* < 0.01 (one-way ANOVA followed by SNK test).

In this study, SM-102, an ionizable lipid used in SpikeVax^®^, was selected as a model lipid material (Figure 1a). The adjuvant activity of the LNPs composed of SM-102 was obtained as the antibody production against ovalbumin (OVA). To exclude the contribution of IVT-mRNA to the adjuvant activity and the number of expressed antigens to the antibody production, empty LNPs were mixed with recombinant ovalbumin (OVA) protein and then administered to the BALB/c mice (Figure 1b). The LNPs also contained distearyol-sn-glycero phosphatidylcholine (DSPC), cholesterol, and PEGylated lipids (DMG-PEG_2000_) [38,39,40]. A lipid composition of SM-102/DSPC/cholesterol/PEG-lipid (50/10/38.5/1.5) was used as the original composition in a SpikeVax^®^, since this composition has been used in vaccines administered to humans and is widely recognized as having vaccine efficacy, albeit with side effects [38,39]. It should be noted that this composition has also been used in other formulations, such as ONPATTRO^®^ and Comirnaty^®^. However, the lipid molecules are different [43,44]. Three sample sets were tested for their adjuvant activity (Figure 1c). In Sample_Set_1, the ratio of SM-102 and cholesterol was varied (LNP_A-D). The LNP_C is equivalent to the lipid composition of the vaccine. Since we first considered that SM-102 is responsible for the activation of adjuvant activity, a sample that replaced all cholesterol with SM-102 was used (LNP_A: SM-102/DSPC/cholesterol/PEG-lipid = 88.5/10/0/1.5). Based on research concerning liposomes, cholesterol is known to have a significant effect on the property of lipid bilayers at around 33.3 mol% [45,46,47]. Therefore, a sample with a cholesterol content lower than 33.3% was also used (LNP_B: SM-102/DSPC/cholesterol/PEG-lipid = 70/10/18.5/1.5). The solubility limit of cholesterol in a phospholipid bilayer is known to be approximately 66% [48]. Therefore, we also set a sample containing cholesterol near the upper limit (LNP_D: SM-102/DSPC/cholesterol/PEG-lipid = 30/10/58.5/1.5). When it was assumed that the cholesterol molecules had dissolved into the LNPs, the solute was considered to be SM-102/DSPC (and additional PEG-lipid). In Sample_Set_2, the ratio of SM-102 and DSPC was fixed, and then the cholesterol content was varied (LNP_C, E–G). In Sample_Set_3, the ratio of SM-102, DSPC, and DMG-PEG_2000_ was fixed, and then the cholesterol content was varied (LNP_C, H–J). Liposomes containing only DSPC, cholesterol, and DMG-PEG2000 were used as a control sample set (Liposome_A-D). As another control, cholesterol nanoparticles dispersed in DMG-PEG_2000_ were also used (dispersed cholesterol; cholesterol/DMG-PEG_2000_ = 98.5/1.5). The particle properties of these samples are summarized in Table 1. Following two administrations with an interval of 14 days, the OVA-specific antibody titer in the serum was evaluated via an ELISA.

The removal of the ionizable lipid revealed a complete disappearance of antibody production (Figure 1d, Liposome_A–D), which is consistent with the results of a previous study reported in the literature [31]. Also, the dispersed cholesterol showed no adjuvant activity. In the case of LNPs, the antibody production did not correlate with the content of ionizable lipids (LNP_A–D) (Figure 1d). The antibody production increased as the ratio of ionizable lipid was decreased by 50% (LNP-C), and it was then decreased when the ionizable lipid density was further decreased to 30% (LNP_D). These results revealed that the adjuvant activity of the LNPs does not simply depend on the dose of ionizable lipid. The antibody production by both Sample_Set_2 (Figure 1e) and Sample_Set_3 (Figure 1f) supported this observation (Figure S1). To exclude the possibility that the variation in antibody production derived from the difference in the degree of physical interaction between LNPs and OVA protein, the empty LNPs and ovalbumin were separately administered to the same location with different timing—the empty LNPs were administered to the skin first, and then the OVA proteins were administered to the same site 6 h later. The production of OVA-specific antibodies through this separate injection showed a trend that was similar to the co-administration experiments of LNP_A-D (Figure S2). This result indicates that the variation in antibody production cannot be explained from the point of view of the difference in the interaction of LNPs and the OVA antigen protein. These observations collectively indicate that the presence of ionizable lipids is at least necessary for the adjuvant effect of the LNPs, but additional factors should be taken into account to explain the adjuvanticity of LNPs.

### 3.2. The Amounts of Ionizable Lipid and Cholesterol Affect the Particle Morphology

**Figure 2:**
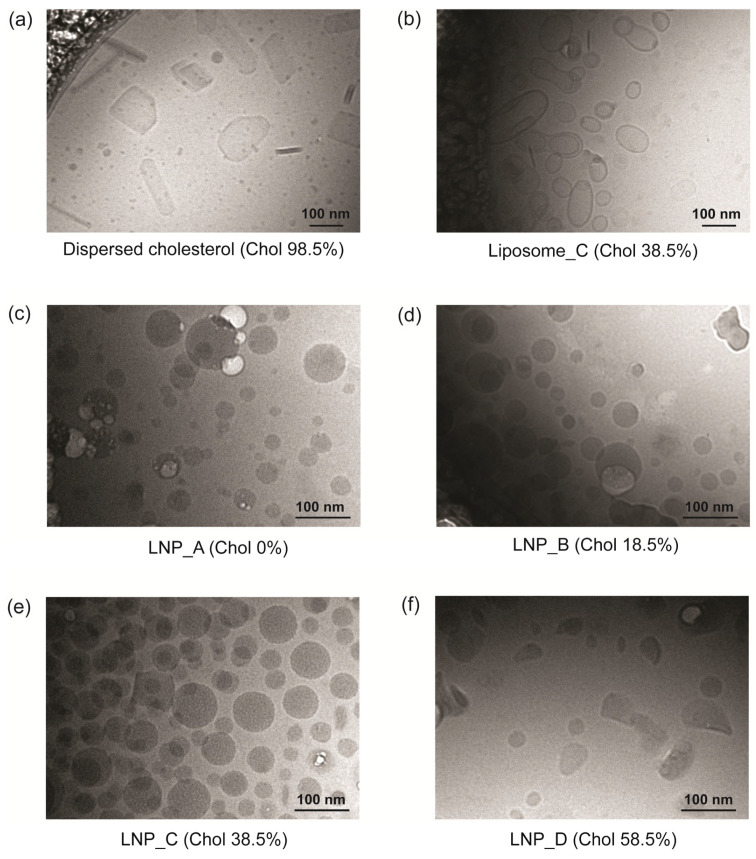
Particle morphology observed via Cryo-TEM. (**a**) Nano-crystal structures of dispersed cholesterol particles. (**b**) Vesicle-like structures of Liposome_C particles. (**c**–**e**) Spherical particles with electron-dense cores of LNP_A, LNP_B, and LNP_C. (**f**) Particles with partially linear edges of LNP_D.

The effect of lipid composition on the morphology of empty LNPs was investigated. A Cryo-TEM observation was conducted for Sample_Set_1 (LNP_A–D), for dispersed cholesterol (cholesterol/DMG-PEG_2000_ = 98.5/1.5), and for Liposome_C (DSPC/cholesterol/DMG-PEG_2000_ = 60/38.5/1.5). The dispersed cholesterol samples showed cholesterol nano-crystals with linear edges (approx. 100 nm in size) (Figure 2a). The Liposome_C particles showed vesicle-like structures with lipid bilayers (Figure 2b). The LNP_A-D particles, however, showed electron-dense droplet-like structures, and the LNP_A–C (0~38.5% cholesterol) particles showed structures that were mainly spherical (Figure 2c–e). The structures of the LNP_D (58.5% cholesterol) particles were not completely spherical and were also dispersed but with partially linear edges, as shown in Figure 2f. The images of each of the samples appear in Figure S3. In combination with the adjuvant activity of each particle (Figure 1d–f), these observations include three findings. First, the removal of ionizable lipids from LNPs changed their structures to those of vesicles, and the corresponding liposomes did not show adjuvant activity. Second, the further removal of phospholipids changed their structure by forming a crystalline-like structure of cholesterol assembly. In this case, an adjuvant effect was not observed. Finally, LNPs containing large amounts of cholesterol partially showed a crystalline-like structure of cholesterol domains that could have a negative effect on the adjuvant activity. Therefore, the droplet-like structures of the LNPs with cholesterol molecules that do not form crystalline-like structures could be considered important for adjuvant activity.

### 3.3. The Intra-Particle State of Cholesterol Varied Depending on Its Amount in the LNPs

**Figure 3:**
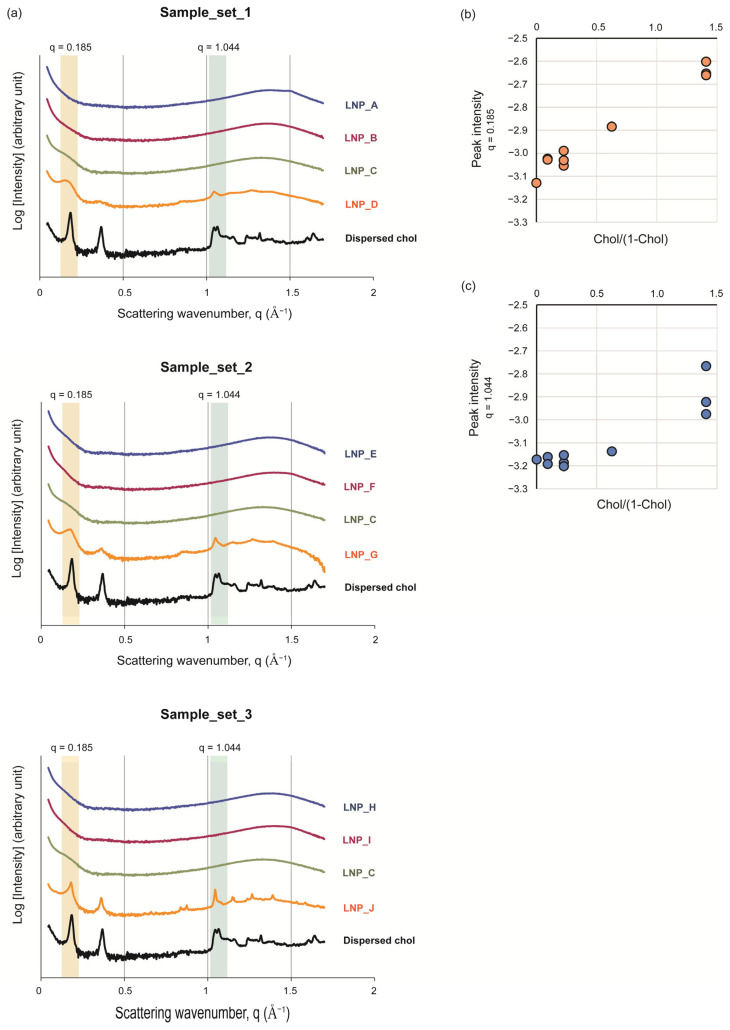
SAXS profiles of dispersed cholesterol and LNPs. (**a**) The SAXS profiles of LNPs in Sample_set_1, Sample_set_2, and Sample_set_3 are summarized. Small-angle peaks at q = 0.185 Å^−1^ and wide-angle peaks at q = 1.044 Å^−1^ of the cholesterol domain appear in the LNPs as the cholesterol content increases. (**b**) The intensity of the small-angle peaks (q = 0.185 Å^−1^) increase linearly with relative cholesterol content. (**c**) The intensity of the wide-angle peaks (q = 1.044 Å^−1^) only increase when the relative cholesterol content reaches a high level.

The intra-particle structures of Sample_Set_1–3 were investigated via a SAXS analysis. Dispersed cholesterol served as a control (Figure 3a). The SAXS charts of Liposome_A–D are summarized in Figure S4. In the case of dispersed cholesterol, peaks were observed in both the small-angle and wide-angle regions. This observation indicated that cholesterol nanocrystals possess both short-distance and long-distance repeated structures. Clear peaks at q = 0.185 Å^−1^ and q = 0.371 Å^−1^ were observed in the small-angle region. In the wide-angle region, a large number of complex peaks were observed, most notably the peak at q = 1.044 Å^−1^. In the LNPs, these three peaks demonstrated a lipid-composition-dependent manner with a linear relationship between the peak intensity at q = 0.185 Å^−1^ and the relative cholesterol content (Figure 3b). Therefore, we suggest that the nanostructures formed with an increasing cholesterol content. In contrast, no peak intensities were detected at q = 0.371 Å^−1^ and q = 1.044 Å^−1^ with cholesterol contents of 0–18.5% as opposed to when a large amount of cholesterol (58.5%) was incorporated (Figure 3a,c). These observations suggest that cholesterol can exist in at least two different states in the LNPs: an intermediate state with a peak at only q = 0.185 Å^−1^ and a crystalline-like state with peaks at q = 0.185 Å^−1^, q = 0.371 Å^−1^, and q = 1.044 Å^−1^.

Drastic changes in the physical properties of cholesterol in the LNPs were also suggested by the circular dichroism (CD) measurements. The spectrum of CD normalized at an absorbance of 204 nm appears in Figure S5a. LNPs without cholesterol showed no CD, whereas the absolute value of the CD was increased as the cholesterol content increased. The LNP_A, LNP_B, and LNP_C particles showed a linear relationship between the normalized CD and the cholesterol ratio. This suggests that the CD values reflected the concentrations of the cholesterol in the dispersions. On the other hand, the LNP_D showed an absolute value of CD that was larger than expected based on the trend of the other LNPs (Figure S5b). It is noteworthy that the peak of the CD of LNP_D differed from that of LNP_A–C. This observation also suggests that the state of cholesterol changes when its content is at a high level.

### 3.4. The Intermediate State of the Cholesterol in LNPs Could Be Important for Adjuvant Activity

**Figure 4:**
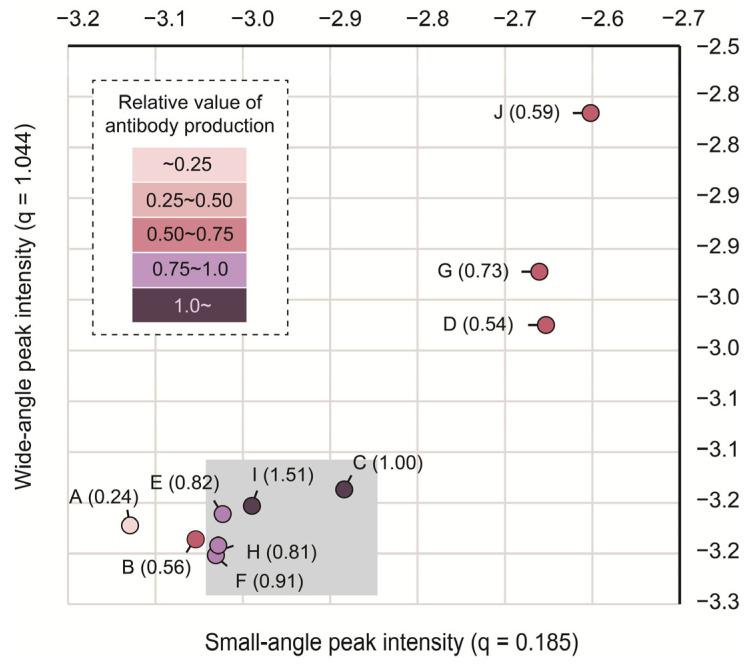
Correlation between the state of cholesterol in LNPs with antibody production. Small- and wide-angle peaks in intensity were plotted against each other and correlated with the antibody production of each LNP. The antibody production values reflect the relative antibody production against LNP_C (1.00). The gray areas (LNP_C, LNP_E, LNP_F, LNP_H, and LNP_I) indicate lipid compositions with high adjuvant activity.

**Figure 5:**
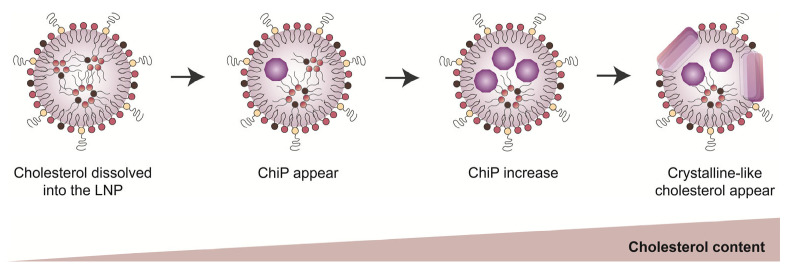
Schematic illustration of the cholesterol-induced phase (ChiP). In cases of low cholesterol content, cholesterol molecules are dissolved in the oil phase made of ionizable lipids. In cases of high cholesterol content, the cholesterol molecules form crystalline-like structures in LNPs. In the intermediate region, an increase in the cholesterol content results in the formulation of a structure with only a short-distance repeat. The broadness of the SAXS peak suggests that the structure might not be static, but instead could be a relatively dynamic structure with fluctuation. These nanostructures are referred to as the cholesterol-induced phase (ChiP) in order to distinguish them from the crystalline-like structures of cholesterol.

The peak intensities at q = 1.044 Å^−1^ and q = 0.185 Å^−1^ are plotted in Figure 4. In this figure, the relative antibody production of each LNP against LNP_C is indicated by colors. When the peak intensity of only q = 0.185 Å^−1^ varied, an increase in the cholesterol content resulted in higher antibody production. On the other hand, at a higher cholesterol content where the peak intensity of q = 1.044 Å^−1^ as well as q = 0.185 Å^−1^ varied, the antibody production tended to decrease. Taking the morphology of LNP_D into account (Figure 2f), this observation again suggests that the formation of a crystalline-like structure of cholesterol reduces the adjuvant activity of LNPs. The intermediate structure that appeared when the cholesterol content was lower did not appear as “crystals” since it showed only a short-distance repeated structure. We suggest that this phase is the cholesterol-induced phase (ChiP) (Figure 5), which could be involved in the adjuvant activity of LNPs.

## 4. Discussion

The number of reports on the adjuvant activity of LNPs in RNA vaccines is growing. The empty LNP that is proprietary to Acuitas Therapeutics has shown the capability of inducing the maturation of monocyte-derived dendritic cells (MDDCs) by regulating the expressions of CD40 and CD83 in human MDDCs [49]. This LNP also promotes the activation of T follicular helper (Tfh) cells and germinal center (GC) B cells, which are important modulators for the B cell response to antigens [31,49]. An optimal LNP composition is also known to increase the RNA uptake by splenic cells [50] and to enhance the activation of innate immune responses by significantly inducing IL-6, CXCL1, CCL2, and CXCL10. Chemokines and cytokines are also involved in the recruitment of monocytes and neutrophils [51]. These observations show that LNPs indeed function as adjuvants in RNA vaccines.

In the present study, the effect that the lipid compositions of LNPs exerted on their adjuvant activity was investigated. To simply evaluate the adjuvant effect of LNPs, a series of empty LNPs with various lipid compositions was co-injected with antigen protein (OVA). Consistent with the citations in previous studies in the literature, the removal of ionizable lipids caused a disappearance of the adjuvant activity in LNPs [31]. DSPC has a phase transition temperature of 55 °C and forms a gel phase at that temperature during formulation and administration. Since the addition of cholesterol is thought to promote the lateral diffusion of DSPC molecules, the addition of cholesterol leads to the phase transition of liposome samples from the gel phase to the liquid-ordered phase [45,47]. However, the liposome samples (Liposome_A–D) did not show adjuvant activity regardless of the amount of cholesterol added. This observation not only suggests that the phase behavior of a simple lipid bilayer had no effect on the adjuvant capacity in our experimental setup, but also that the presence of an ionizable lipid is essential for adjuvant activity. If ionizable lipids directly stimulate inflammatory signals, the adjuvant activity of LNPs is expected to increase when the ionizable content of lipids increase, but the results were completely the opposite. Where antibody production was expected to be induced when the ionizable lipid content was reduced, the antibodies were instead induced when the cholesterol content was increased (Figure 1d). It is noteworthy that the nearly maximum antibody production was exhibited when the lipid composition was adjusted to that employed in the Spikevax^®^. Thus, it is plausible to assume that the mechanisms proposed in this study using empty LNPs could be extrapolated to mRNA-LNPs.

The Cryo-TEM images of the dispersed cholesterol indicated that cholesterol molecules with the DMG-PEG_2000_ form nanocrystal-like structures with sharp edges. Cholesterol molecules form bilayer structures due to their amphiphilic nature [52,53,54], and the thickness of a cholesterol bilayer is 33.9 Å. The appearance of a SAXS peak at 0.186 Å^−1^ corresponded to a Bragg distance of 33.9 Å, which clearly suggests that dispersed cholesterol forms similar bilayer structures. In crystalline-like cholesterol, the bilayer structures are stacked to form a long-distance repeated structure. The SAXS peak at 0.371 Å^−1^ is considered to be the second-order diffraction in the order of the stacked structures. It should also be noted that the wide-angle region of the SAXS chart of the dispersed cholesterol samples corresponds well to the powder X-ray diffraction of cholesterol crystals [55].

In contrast to dispersed cholesterol, the cholesterol molecules in LNPs are expected to occur in at least two states. At a higher cholesterol content of approximately 60%, peaks appeared at q = 0.371 Å^−1^ and q = 1.044 Å^−1^. This indicates that a portion of cholesterol forms stacked bilayer structures when dispersed. In other words, in LNPs with high cholesterol contents, crystalline-like domains form the edged structures that we observed in Cryo-TEM images (Figure 2f). On the other hand, with a lower cholesterol content (below 40%), only a broad peak at q = 0.185 Å^−1^ appeared in comparison with the peak when dispersed cholesterol was used. The absence of the second-order diffraction and the broadness of the peak suggests that the cholesterol molecules in this structure have high mobility and/or a thickness distribution. Based on these observations, we hypothesize that the cholesterol particles in LNPs form an intermediate bilayer-like structure, which we refer to as the ChiP in order to distinguish it from the crystalline-like structures of cholesterol. When the cholesterol content exceeds a certain limit, a portion of the ChiP could be stacked in order to form a crystalline-like structure. The most important finding here is that the degree of antibody production is closely related to the intra-particle formulation of the ChiP structure.

The environment that surrounds the dispersed cholesterol molecules differs from that in the ChiP structure. In the case of dispersed cholesterol, cholesterol molecules are dispersed in the aqueous phase. Thus, the cholesterol is mutually assembled to form a crystal nucleus in order to minimize the contact area with the water phase. On the other hand, in LNPs, the cholesterol molecules are dispersed in the lipid-rich hydrophobic environment since the ionizable lipids at a neutral pH form a droplet-like structure. Under this environment, the cholesterol forms an intermediate bilayer-like structure (ChiP) via self-assembly when the cholesterol content is approximately 40% (38.5% in Figure 3: LNP_C). This is because the removal of ionizable lipids causes a complete disappearance of the adjuvant activity, which is likely due to the disappearance of the ChiP structure, since the oil phases that tend to stabilize the ChiP structure are diminished by the change in the structure in the lipid bilayer. Quantitative discussion remains difficult since the intra-particle distribution of lipid components is passively affected by the composition of LNPs. Neutron scattering experiments suggest that LNPs have a core–shell type structure [56]. Phospholipids, such as DSPC, are exclusively present on the surface shell. The core is composed mainly of ionizable lipids and cholesterol. Since the replacement of cholesterol with its derivatives causes changes in the morphology and rigidity of LNPs [57], cholesterol is thought to be present in both the core and the shell. A general method to evaluate/calculate the relative proportions of cholesterol in the core and shell of LNPs remains to be established. Therefore, a quantitative discussion concerning the exact phase behavior of those regions is limited. Developing a method to quantify the intra-particle distribution would shed light on the immunological properties of LNPs.

In the present study, we found that the formulation of the ChiP in LNPs is closely related to antibody production. However, the relationship between the LNP-triggered innate immunity and the induction of antibody production remains unclear. When focusing on chemokine/cytokine production by LNP_C, the levels of IFN-γ, CXCL1, CCL2, CXCL10, and IL6 tend to reflect a dose dependency (Figure S6). This observation shows that empty LNPs actually induce an inflammatory reaction following administration. However, the chemokine/cytokine production of LNPs with different lipid compositions do not show a correlation with their antibody production (Figure S7). These data suggest that the systemic cytokine/chemokine levels cannot be an index for the adjuvant activity of LNPs.

Finally, the mechanism for the ChiP-triggered activation of innate immunity remains to be elucidated. Cholesterol crystals are known to cause inflammatory cell death by injuring the plasma membrane of the cells. Contact between bone-marrow-derived macrophages (BMDMs) and cholesterol crystals induces a necrotic membrane rupture of the cells. This event was found to be independent of cellular signaling and caused solely by physical contact with cholesterol crystals [58]. A mechanism of this inflammatory cell death was hypothesized as follows: When a cholesterol crystal makes contact with the plasma membrane, it absorbs the cholesterol particles on the cell membrane, which causes further crystal growth. The decrease in the membrane integrity caused by a reduction in the cholesterol content in the bio-membrane finally results in cell death. It is assumed that the ChiP may also affect the cholesterol content in the plasma membrane, and the release of damage-associated molecular patterns (DAMPs) may activate innate immunity.

## 5. Conclusions

In this study, we proposed that the activation of humoral immunity is closely related to the degree of the formation of nano-sized cholesterol structures rather than to the content of ionizable lipids. This intermediate state of cholesterol is herein referred to as the ChiP in order to distinguish it from the crystalline-like structures of cholesterol. The role of an ionizable lipid is to form a lipid droplet structure that is suitable for the stabilization of the ChiP structure. Controlling the assembly of cholesterol in LNPs could be a useful strategy to modify the immune-stimulative activity of LNPs.

## Figures and Tables

**Table 1:** Lipid composition and physicochemical properties of empty particles.

Particle	Composition (%)				Size (d.nm)	PdI	ZP (mV)
	SM-102	DSPC	Chol	PEG2K			
Dispersed chol.	0	0	98.5	1.5	147 ± 10.2	0.23 ± 0.0	−4.2 ± 1.2
Liposome_A	0	98.5	0	1.5	183 ± 4.9	0.53 ± 0.1	0.7 ± 0.4
Liposome_B	0	80	18.5	1.5	178 ± 6.4	0.36 ± 0.1	0.1 ± 0.1
Liposome_C	0	60	38.5	1.5	137 ± 11.9	0.30 ± 0.1	−3.1 ± 1.5
Liposome_D	0	40	58.5	1.5	108 ± 8.0	0.26 ± 0.0	−6.6 ± 1.1
Sample_set_1							
LNP_A	88.5	10	0	1.5	113 ± 2.0	0.11 ± 0.0	6.0 ± 0.9
LNP_B	70	10	18.5	1.5	99 ± 1.6	0.12 ± 0.0	1.8 ± 0.9
LNP_C	50	10	38.5	1.5	89 ± 2.5	0.13 ± 0.0	−0.4 ± 0.5
LNP_D	30	10	58.5	1.5	99 ± 1.8	0.16 ± 0.0	−0.3 ± 0.2
Sample_set_2							
LNP_E	75	15	8.5	1.5	90 ± 6.7	0.15 ± 0.0	0.5 ± 0.6
LNP_F	66.7	13.3	18.5	1.5	86 ± 4.2	0.15 ± 0.0	−0.5 ± 0.7
LNP_C	50	10	38.5	1.5	89 ± 2.5	0.13 ± 0.0	−0.4 ± 0.5
LNP_G	33.3	6.7	58.5	1.5	91 ± 3.8	0.24 ± 0.1	−2.6 ± 0.9
Sample_set_3							
LNP_H	74.4	14.9	8.5	2.2	92 ± 4.1	0.16 ± 0.0	5.6 ± 1.4
LNP_I	66.2	13.3	18.5	2.0	93 ± 5.5	0.21 ± 0.0	0.6 ± 0.4
LNP_C	50	10	38.5	1.5	89 ± 2.5	0.13 ± 0.0	−0.4 ± 0.5
LNP_J	33.7	6.8	58.5	1.0	105 ± 5.2	0.24 ± 0.0	−4.5 ± 1.2

Size, PdI, and ZP were measured using a Zetasizer Nano ZS. Mean ± SD (*n* = 3).

## Data Availability

The data presented in this study are available on request from the corresponding authors.

## Supplementary Materials

The following supporting information can be downloaded at https://www.mdpi.com/article/10.3390/pharmaceutics16020181/s1. S1 is a detailed list of materials used in the study. S2 describes the methods for Supplementary Figures (S2-1, circular dichroism measurement; S2-2, chemokine/cytokine measurement), and S3 features the Supplementary Figures: Figure S1. Correlation of lipid content and antibody production; Figure S2. Antibody production after separate administration of LNPs and antigens; Figure S3. Additional images of particle morphology observed using Cryo-TEM; Figure S4. The SAXS profile of Liposomes_A-D; Figure S5. Circular dichroism (CD) spectra; Figure S6. Dose-dependent chemokine/cytokine production; and Figure S7. Chemokine/cytokine production of different nanoparticles. Table S1. Detailed list of materials used in the study.
