# Supplementary material for: The Effect of Cholesterol Content on the Adjuvant Activity of Nucleic-Acid-Free Lipid Nanoparticles
Source: Pharmaceutics. 2024 Jan 26;16(2):181. doi: 10.3390/pharmaceutics16020181 (PMC10893020; doi:10.3390/pharmaceutics16020181)
Supplement: Supplementary file 1 [file pharmaceutics-16-00181-s001.zip › pharmaceutics-2790348-supplementary.pdf]

Supplementary Materials

Pharmaceutics

Title:

The Effect of Cholesterol Content on the Adjuvant Activity of Nucleic-Acid-Free Lipid Nanoparticles

Authors:

Jessica Anindita[1,2,†], Hiroki Tanaka[1,3,*,†], Takuma Yamakawa[2], Yuka Sato[2], Chika Matsumoto[1], Kota Ishizaki[2], Taiji Oyama[4], Satoko Suzuki[5], Keisuke Ueda[6], Kenjirou Higashi[6], Kunikazu Moribe[6], Kasumi Sasaki[7], Yumika Ogura[7], Etsuo Yonemochi[7], Yu Sakurai[1], Hiroto Hatakeyama[2], and Hidetaka Akita[1,3,*]

Affiliations

Jessica Anindita [1,2,†], Hiroki Tanaka [1,3,*,†], Takuma Yamakawa [2], Yuka Sato [2], Chika Matsumoto [1], Kota Ishizaki [2],
Taiji Oyama [4], Satoko Suzuki [5], Keisuke Ueda [6], Kenjirou Higashi [6], Kunikazu Moribe [6], Kasumi Sasaki [7],
Yumika Ogura [7], Etsuo Yonemochi [7], Yu Sakurai [1], Hiroto Hatakeyama [2] and Hidetaka Akita [1,3,*]

[1] Laboratory of DDS Design and Drug Disposition, Graduate School of Pharmaceutical Sciences, Tohoku University, 6-3 Aoba Aramaki, Aoba-ku, Sendai City 980-8578, Miyagi, Japan
[2] Laboratory of DDS Design and Drug Disposition, Graduate School of Pharmaceutical Sciences, Chiba University, 1-8-1 Inohana, Chuo-ku, Chiba City 260-0856, Chiba, Japan
[3] Center for Advanced Modalities and DDS, Osaka University, Suita 565-0871, Osaka, Japan
[4] Sales Division, JASCO Corporation, 2967-5 Ishikawa, Hachioji City 192-8537, Tokyo, Japan; taiji.oyama@jasco.co.jp
[5] Applicative Solution Lab Division, JASCO Corporation, 2967-5 Ishikawa, Hachioji City 192-8537, Tokyo, Japan
[6] Laboratory of Pharmaceutical Technology, Graduate School of Pharmaceutical Sciences, Chiba University, 1-8-1 Inohana, Chuo-ku, Chiba City 260-0856, Chiba, Japan; keisuke@chiba-u.jp (K.U.)
[7] Department of Physical Chemistry, School of Pharmacy and Pharmaceutical Sciences, Hoshi University, 2-4-41 Ebara, Shinagawa-ku, Shinagawa City 142-8501, Tokyo, Japan
***** Correspondence: hiroki.tanaka.e1@tohoku.ac.jp (H.T.); hidetaka.akita.a4@tohoku.ac.jp (H.A.); Tel.: +81-22-795-6831 (H.T. & H.A.)
† These authors contributed equally to this work.

Contents

S1. Detailed list of materials used in the study.

S2. Methods for supplementary figures

- **S2-1. Circular dichroism measurement**
- **S2-2. Chemokine/cytokine measurement**

S3. Supplementary figures

- **Figure S1. Correlation of lipid content and antibody production**
- **Figure S2. Antibody production after separate administration of LNPs and antigens**
- **Figure S3. Additional images of particle morphology observed using Cryo-TEM**
- **Figure S4. The SAXS profile of Liposomes_A-D**
- **Figure S5. Circular dichroism (CD) spectra**
- **Figure S6. Dose-dependent chemokine/cytokine production**
- **Figure S7. Chemokine/cytokine production of different nanoparticles**

Supplementary Table S1. Detailed list of materials used in the study

Reagent	Size	Manufacturer	Product number
SM-102	100 mg	Cayman Chemical	33474
COATSOME MC-8080 (DSPC; 1,2-Distearoyl-sn-glycero-3-phosphocholine)	1 g	NOF CORPORATION	MC-8080
Cholesterol	5 g	Sigma Aldrich	C8667-5G
DMG-PEG (1,2-dimyristoyl-rac-glycero-3-methoxypolyethylene glycol) 2000	1 g	NOF CORPORATION	GM-020
Sodium Acetate (CH_3COONa)	500 g	nacalai tesque	C31118-75
MES (2-(*N*-morpholino)ethanesulfonic acid)	100 g	nacalai tesque	02442-44
HEPES (4-(2-hydroxyethyl)-1-piperazineethanesulfonic acid)	500 g	Dojindo	342-01375
D-PBS (-)	500 mL	nacalai tesque	14249-24
99.5% Ethanol	500 mL	nacalai tesque	14713-95
UltraPure ™ DNase/RNase-Free Distilled Water (DDW)	500 mL	Invitrogen	10977-023
Albumin from chicken white egg	250 mg	Sigma Aldrich	A2512-250MG
Polyoxyethylene Sorbitan Monolaurate (Tween 20)	500 g	nacalai Tesque	35624-15
Fetal Bovine Serum (FBS)	500 mL	Gibco	10270
Goat anti-Mouse IgG-Fc Fragment Antibody HRP Conjugated	1 mL	Bethyl Laboratories	A90-131P
TMB Solution	100 mL	Merck Millipore	CL07-100ML
1mol/l Sulfuric Acid (H_2SO_4)	500 mL	nacalai Tesque	95626-06
Sodium Hydrogen Carbonate ($NaHCO_3$)	500 g	nacalai Tesque	31212-25
Sodium Carbonate (Na_2CO_3)	500 g	nacalai Tesque	31310-35
LEGENDplex™ Mouse Anti-Virus Response Panel (13-plex) with V-bottom plate	100 tests	BioLegend	740622
Isoflurane Inhalation Anesthetic Solution	250 mL	Viatris	1119701G1092
AmiconUltra-4-100K Centrifugal Units	4 mL volume	Merck	UFC810096
AmiconUltra-4-100K Centrifugal Units	15 mL volume	Merck	UFC910096

S2. Methods for supplementary figures

S2-1. Circular dichroism (CD) measurement

LNP_A-D were prepared as described in the main text. The final concentration of samples was 6.6 mM of total lipid. Circular dichroism of these samples was measured using a J-1500 Circular Dichroism Spectrophotometer (JASCO Corporation, Japan). Parameters were as follows: measured range = 192-280 nm, scanning ratio = 50 nm/min, response = 4 sec, band width = 1 nm, accumulations = 4 times, and repeat = 3 times. The obtained CD spectrum was normalized by absorbance at 204 nm.

S2-2. Chemokine/cytokine measurement

Dispersed cholesterol particles, Liposome_C and LNP_A-D, were administered to BALB/c mice via s.c. injection (back of neck). Six hours after the injection, the blood was collected from the vena cava inferior using a 1 mL syringe and a 26G needle, and it then was moved to a 1.5 mL Eppendorf tube. The blood serum was obtained by incubating the blood at room temperature (RT) for 2 hours, followed by centrifugation (4 ^{o}C, 2,000G, 10 min). The chemokine/cytokine quantification was done using a LEGENDplex™ Mouse Anti-Virus Response Panel (13-plex) with a V-bottom Plate kit (#740622, BioLegend, California, US), following the manufacturer’s instructions. These data were collected by NovoCyte Flow Cytometer Systems and analyzed using the Data Analysis Software Suite for LEGENDplex™. The chemokine/cytokine levels were determined from the mean fluorescence intensity (MFI) values.

Supplementary Figure S1

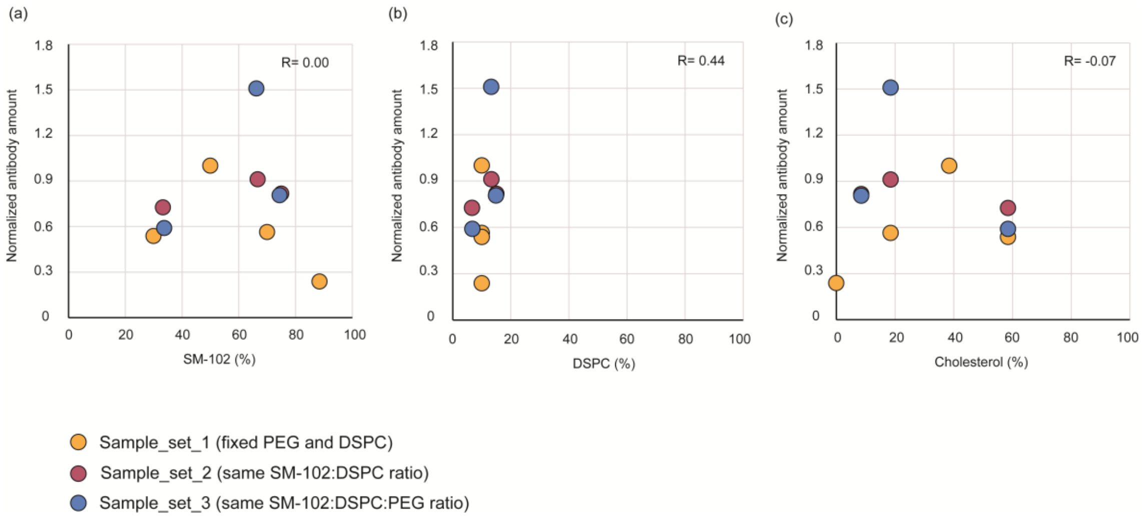

Figure S1. Correlation of lipid content and antibody production: Antibody production 14 days after boost vaccination correlated with **(a)** ionizable lipid SM-102, **(b)** DSPC, and **(c)** cholesterol content within the LNP. No significant correlation was found between antibody production and lipid content.

Supplementary Figure S2

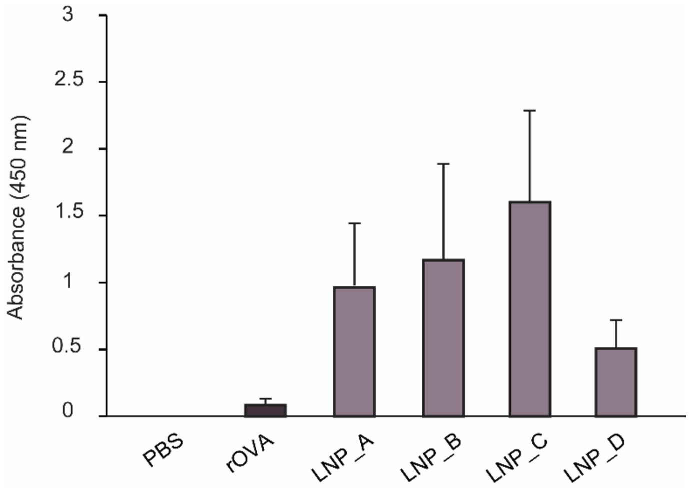

Figure S2. Antibody production after separate administration of LNPs and antigens: The antibody production of LNP_A-D administered separately with recombinant OVA proteins is shown. Doses of 200 nmol of empty-LNPs were administered subcutaneously (s.c.) into BALB/c mice, followed by the administration (s.c.) of 10 µg of rOVA 6 hours later. The treatment was performed twice with a 14-day interval. Later, the blood of each mouse was collected, and the serum was used for anti-OVA total IgG quantification by enzyme-linked immunosorbent assay (ELISA). The antibody production of LNPs did not depend on their ionizable lipid content. Mean ± SD (*n* = 1-5).

Supplementary Figure S3

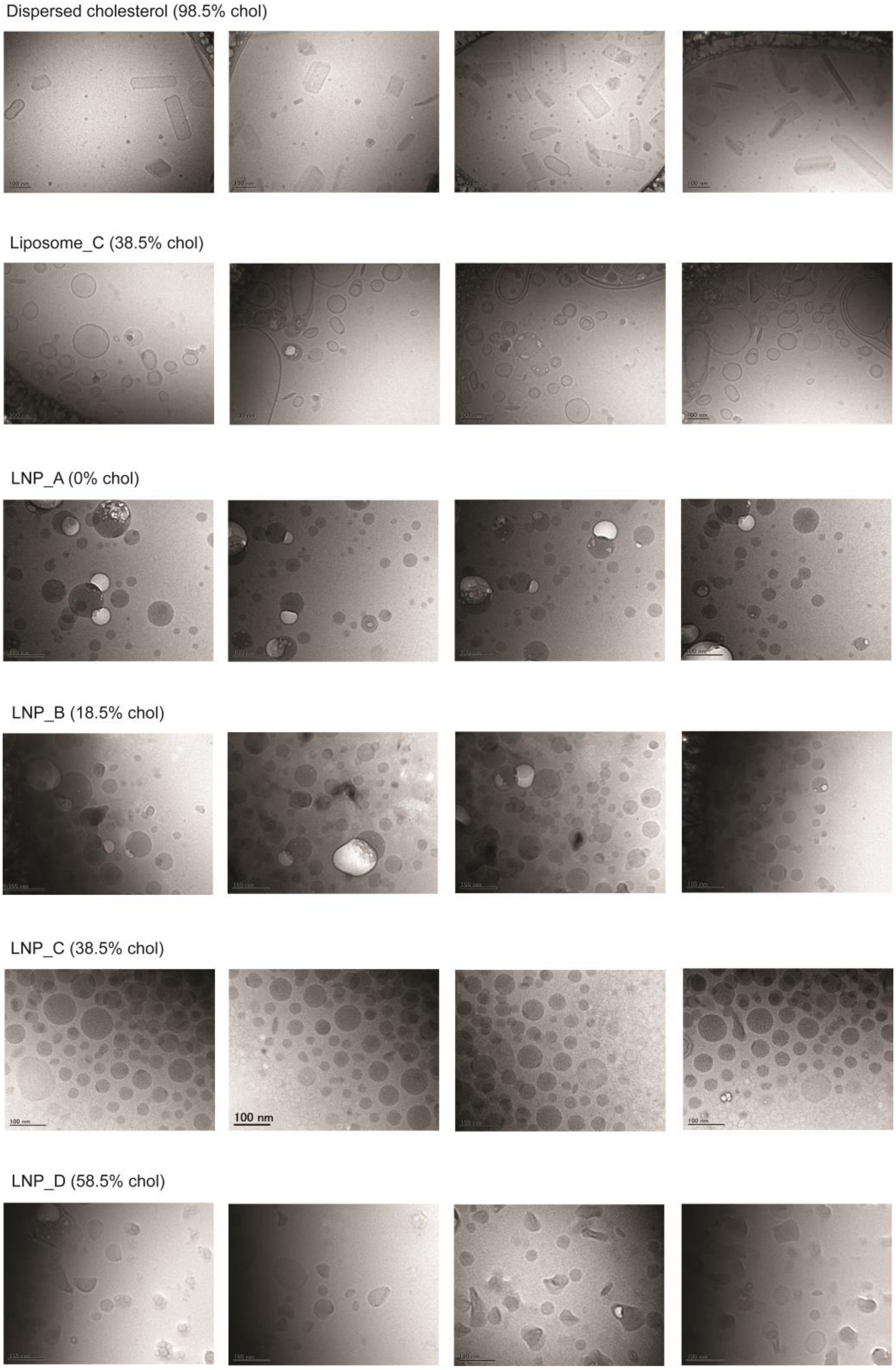

Figure S3. Additional images of particle morphology observed using Cryo-TEM: Additional Cryo-TEM images of dispersed cholesterol, Liposome_C, LNP_A, LNP_B, LNP_C, and LNP_D are shown. Each bar indicates 100 nm.

Supplementary Figure S4

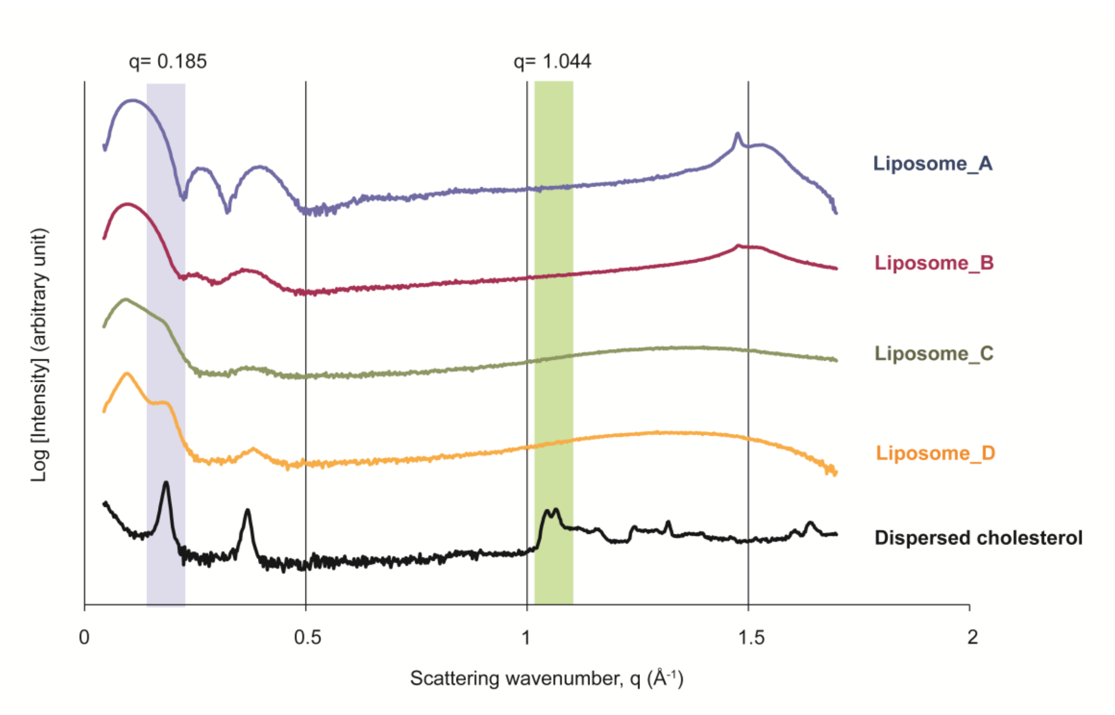

Figure S4. The SAXS profile of Liposomes_A-D: The SAXS profiles of Liposome_A-D are shown. The SAXS profile of dispersed cholesterol particle is included for comparison.

Supplementary Figure S5

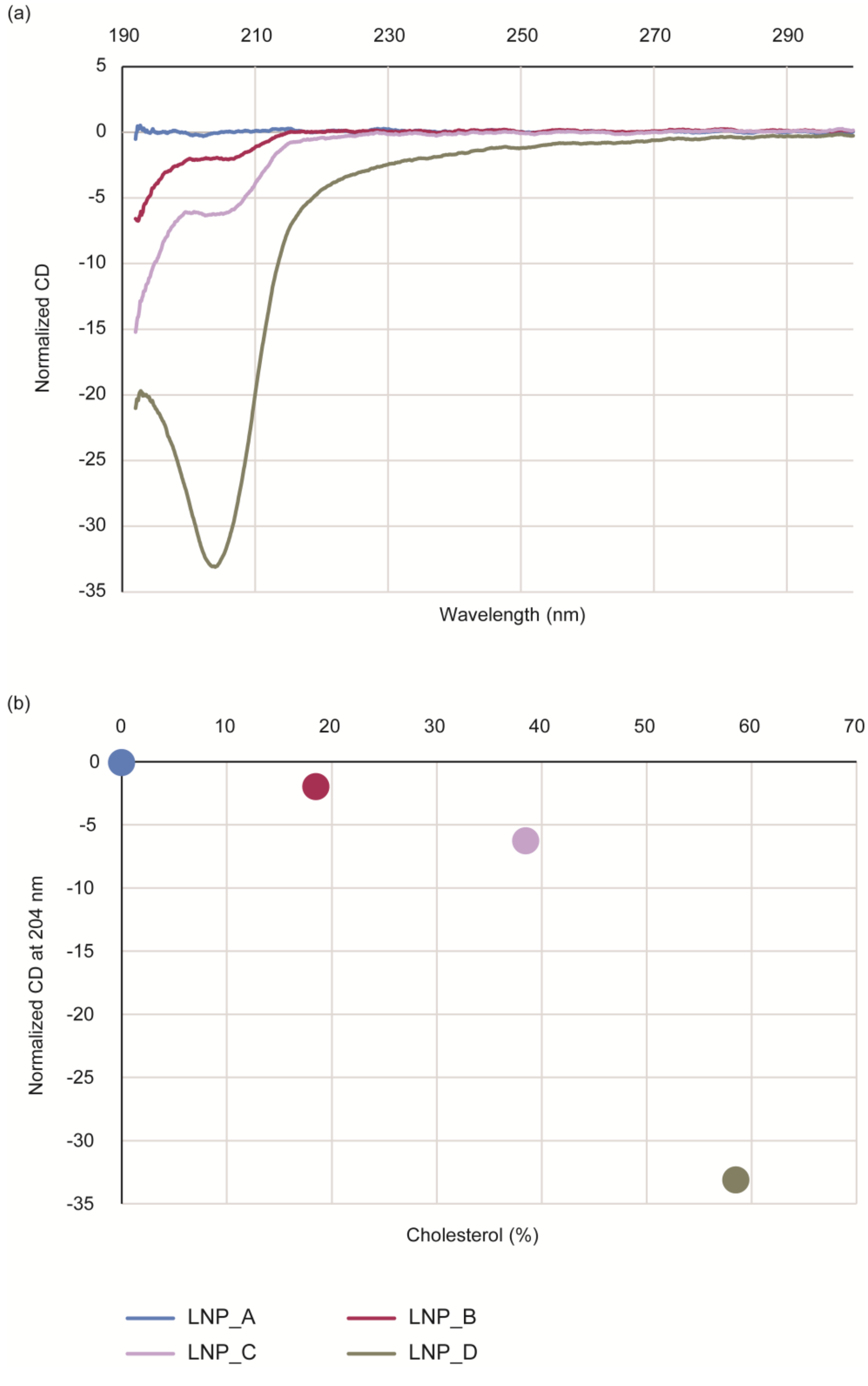

Figure S5. Circular dichroism (CD) spectra: (a) The CD spectra of LNP_A-D normalized at an absorbance wavelength of 204 nm; **(b)** The CD of LNP_A-C show a linear relationship with cholesterol content, while that of LNP_D shows a large absolute value compared with that of the other LNPs.

Supplementary Figure S6

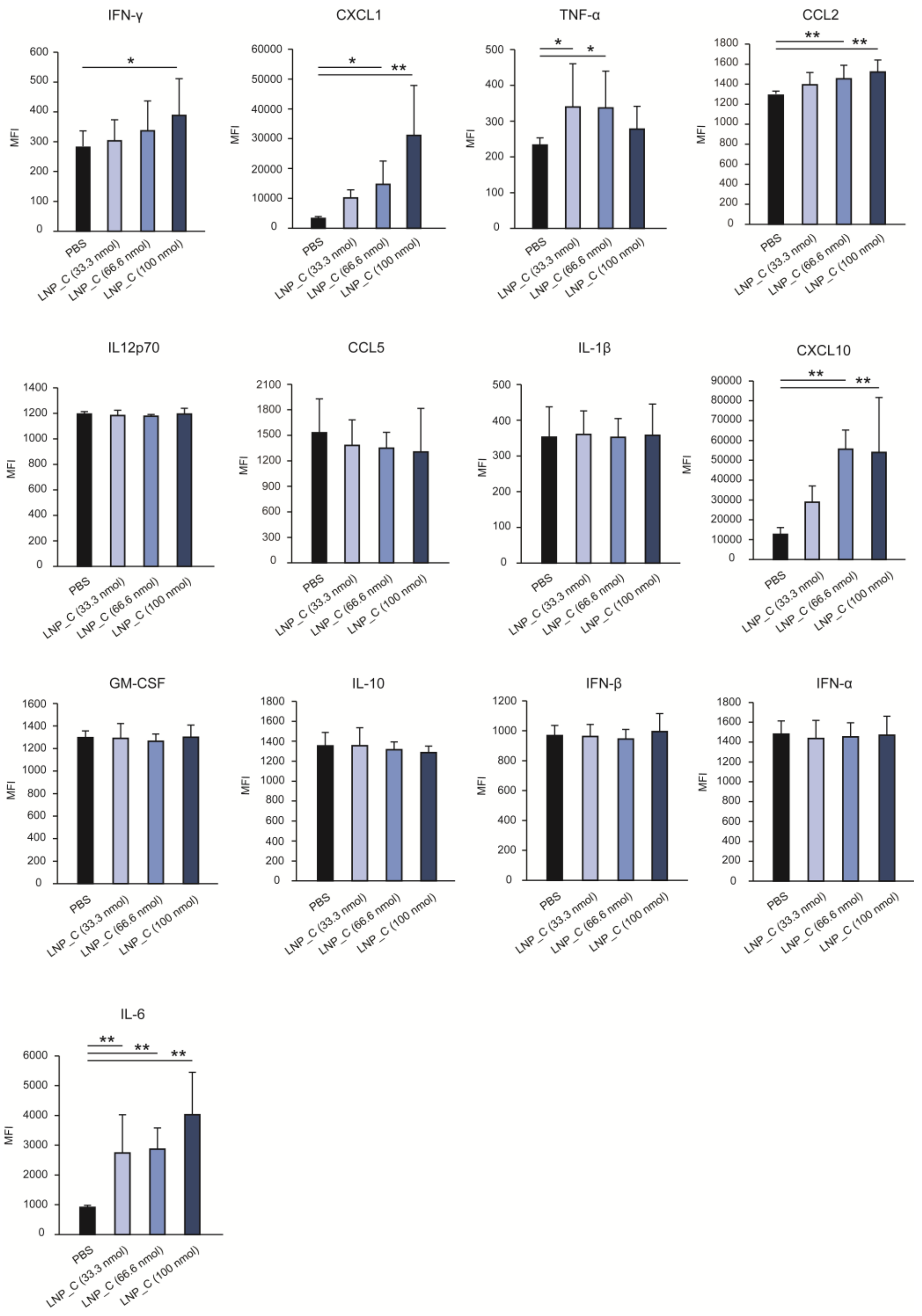

Figure S6. Dose dependent chemokine/cytokine production: The chemokines/cytokines induced by LNP_C are shown with doses of 33.3 nmol, 66.6 nmol, and 100 nmol of total lipid concentration. The respective doses (33.3, 66.6, 100 nmol) of empty-LNPs were administered subcutaneously to the BALB/c mice. After 6 hours, the blood serum was collected and used to quantify the chemokine/cytokine levels. Mean with SD (*n* = 3), * p<0.05, ** p<0.01 (one-way ANOVA followed by Bonferroni against PBS).

Supplementary Figure S7

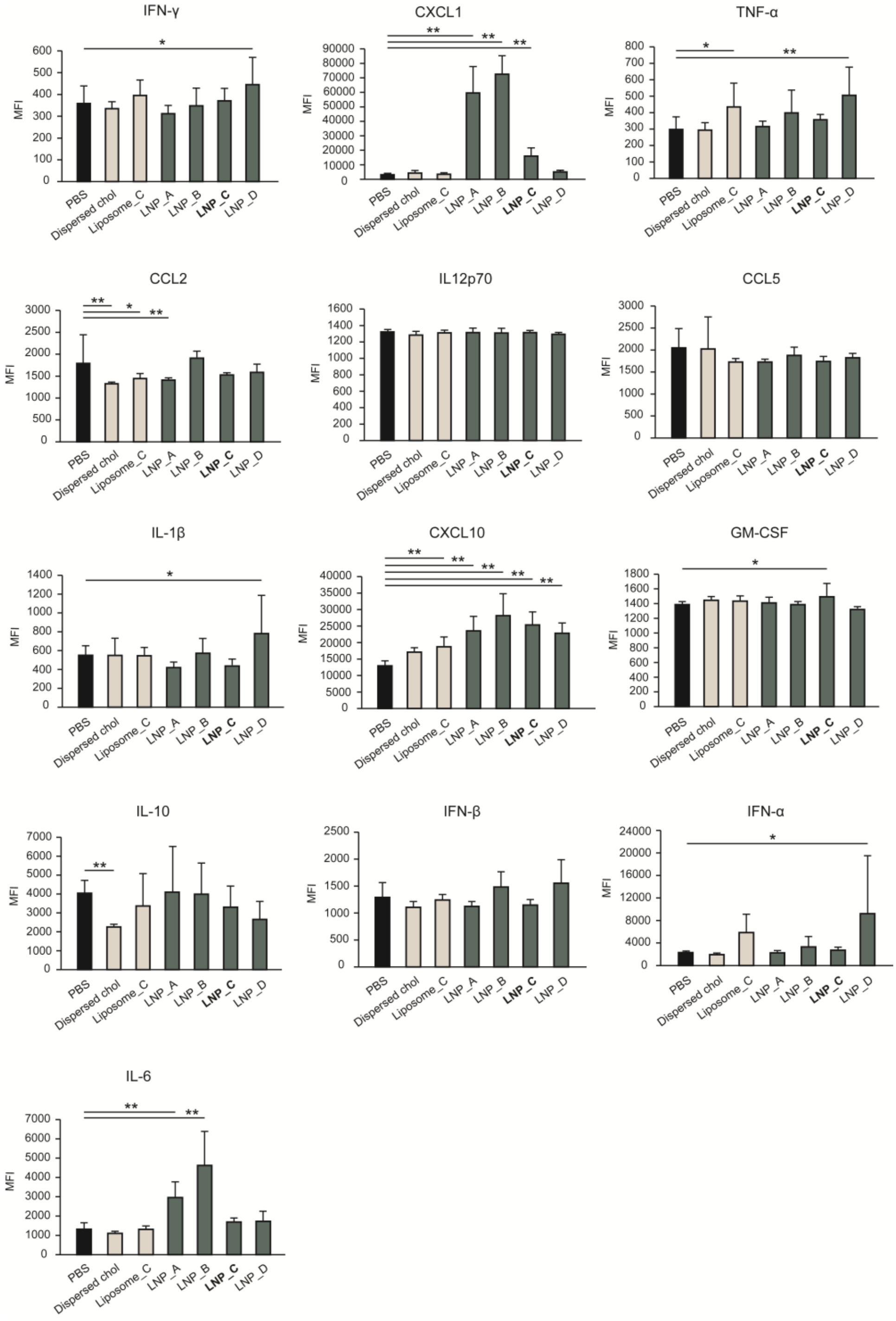

Figure S7. Chemokines/cytokines production of different nanoparticles: The chemokines/cytokines induced by dispersed cholesterol, Liposome_C, and LNP_A-D are shown. Doses of 100 nmol of empty LNPs were administered subcutaneously to the BALB/c mice; after 6 hours, the blood serum was collected and used to quantify the chemokine/cytokine levels. Mean with SD (n = 3), * p<0.05, ** p<0.01 (one-way ANOVA followed by Bonferroni against PBS).
